# AMBER parameters and topology data of 2-pentylpyrrole adduct of arginine with 4-hydroxy-2-nonenal

**DOI:** 10.1016/j.dib.2020.105294

**Published:** 2020-02-19

**Authors:** Antistio Alviz-Amador, Rodrigo Galindo-Murillo, Humberto Pérez-González, Erika Rodríguez-Cavallo, Ricardo Vivas-Reyes, Darío Méndez-Cuadro

**Affiliations:** a Analytical Chemistry and Biomedicine Group, Faculty of Exact and Natural Sciences, Campus of San Pablo, University of Cartagena, First Floor Lab.109. Cra. 50 #24-120, PA 130015, Cartagena, Colombia; b Department of Medicinal Chemistry, Skaggs Pharmacy Research Building, University of Utah, 257 1400 E, Salt Lake City, UT 84112, USA; c Analytical Chemistry and Biomedicine Group, Exact and Natural Sciences Faculty, Campus of Zaragocilla, Ancient Building CREAD, University of Cartagena, Lab 103. Cra. 50 #24-120, PA 130015, Cartagena, Colombia; d Grupo de Química Cuántica y Teórica, Faculty of Exact and Natural Sciences, Campus of San Pablo, University of Cartagena, Second Floor # 202. Cra. 50 #24-120, PA 130015, Cartagena, Colombia

**Keywords:** AMBER, Gaff, Force field parameterization, Quantum-mechanics, Molecular dynamics, Geometry optimization, Validation, Protein carbonylation

## Abstract

The data described here supports a part of the research article “Effect of 4‑HNE Modification on ZU5-ANK Domain and the Formation of Their Complex with β‑Spectrin: A Molecular Dynamics Simulation Study” [1]. Dataset on Gaff force field parameters of AMBER is provided for the non-standard arginine resulting of reaction with 4-hydroxy-2-nonenal (4-HNE), the major secondary product of lipids peroxidation. Arg-HNE 2-pentilpyrrole adduct is part of the 4-hydroxyalkenals described in various physiopathological disorders related to increased oxidative stress. Data include a framework for derivation of missing bonds, angles and dihedral parameters for modified arginine, alongside optimized partial charges derived with Restrained Electrostatic Potential (RESP) method and the new force field parameters obtained by quantum mechanicals methods (QM) using Hartree-Fock (HF)/6 - 31G** level of theory. Benchmark as a graphics tutorial summary steps to obtained new parameters and the validation of non-standard amino acids is presented. The new residue constructed is put available to the scientific community to perform molecular dynamics simulations of modified 4-HNE proteins on arginine residue and complete the set of data parameters for nucleophilic residues with this reactive aldehyde ADDIN EN.CITE ADDIN EN.CITE.DATA [2]. Data that could be used for the researchers interested in the role of protein oxidation as mediator in cellular pathophysiological.

**Table:** Specifications Table

Subject	*Biochemistry, Biophysics*
Specific subject area	*Computational Biochemistry, Computational Biophysics*
Type of data	*Figures and tables*
How data were acquired	*Quantum Mechanics (QM), Molecular Dynamics(MD), Software used: Gaussian 09 for QM, AMBER (pmemd) for MD*
Data format	*Raw and analyzed.*
Parameters for data collection	The 2-pentylpyrrole adduct produced by reaction between 4-Hydroxy-2-nonenal (4-HNE) and arginine was built and optimized in Gaussian D.09 version. Charges, missing bonds, angles, and dihedral angles parameters were constructed with Amber Tools 16. Missing bonds, angles, dihedral parameters and constants of 4-HNE-Arginine were calculated using parmcal
Description of data collection	Computational calcules with Theorical level Hartree-Fock HF/6 - 31G** for QM and Gaff2 force field and ff14SB force field for MD. MD simulations were immersed in a cube of TIP3P water at 300 K and 1 bar. Values of **root-mean-square deviation** (RMSD).
Data source location	*Cartagena, Colombia, Facultad de Ciencias Farmacéuticas and Facultad de Ciencias Exactas y Naturales.*10°23′58.75°30′09., Cl. 6 #3″N, Cartagena, Bolívar.
Data accessibility	*Data are supplied with this article.* Parameter files will be available http://research.bmh.manchester.ac.uk/bryce/amber/
Related research article	Antistio Anibal Alviz-Amador, Rodrigo Galindo-Murillo, Humberto Perez-Gonzalez, Erika Rodriguez-Cavallo, Ricardo Vivas-Reyes and Dario Mendez-Cuadro. Effect of 4-HNE modification on ZU5-ANK domain and the formation of their complex with β-spectrin: A Molecular dynamics simulation study [1]. https://doi.org/10.1021/acs.jcim.9b00772

**Table:** 

**Value of the Data** • Dataset of new AMBER force field parameters are provided to perform Molecular Dynamics Simulation of 4-HNE carbonylated proteins with 2-pentylpyrrole adduct on arginine residues. • A benchmark framework for constructing, parameterizing, optimizing and validating of the new non-standard 4HNE-arginine pyrrole adduct is now available. • Our data can be used to modify, simulate and evaluate by molecular dynamic simulation the effects of 4-HNE carbonylation on arginine over any protein system.

## 1 Data Description

**Fig. 1:**
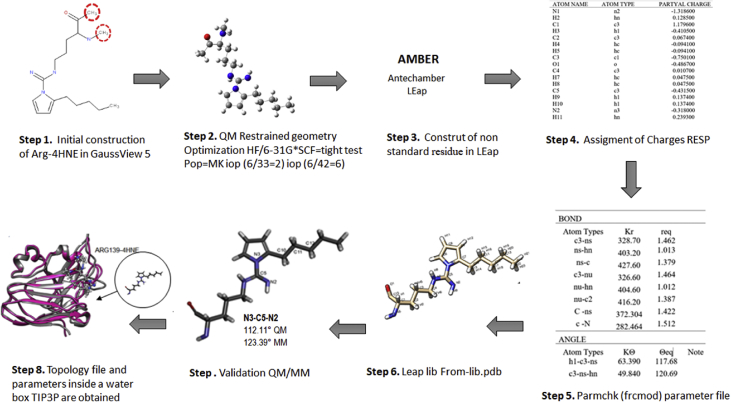
Framework for initial force field parameters and topology of the arginine adduced with 4-HNE.

**Fig. 2:**
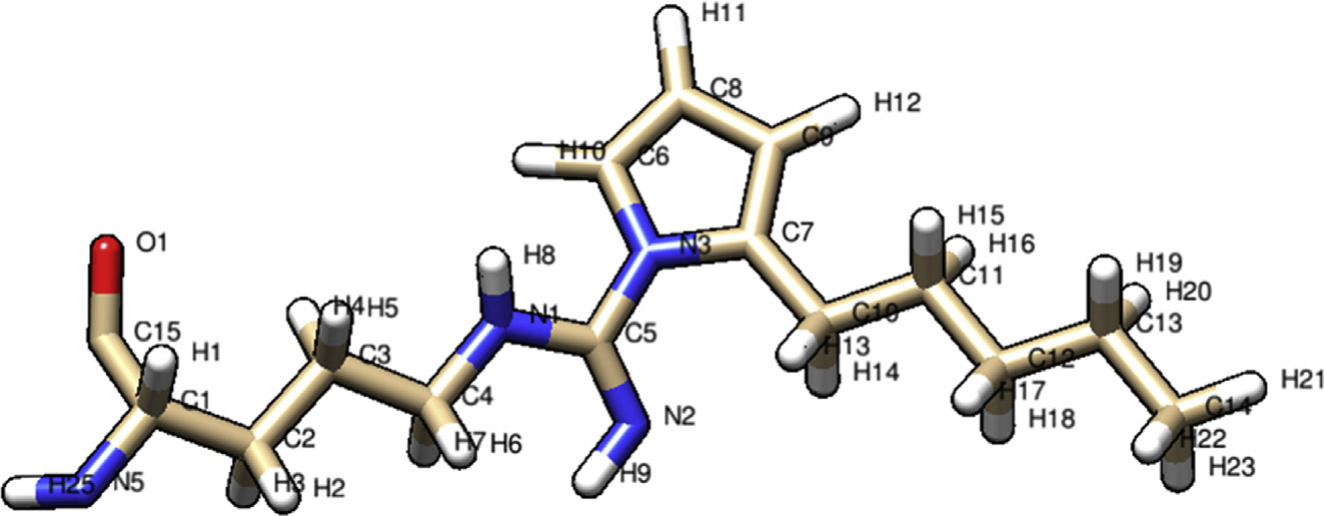
**Optimized structure of 2-Pentyl-pyrroleadduct ARG-4HNE.** Figure was obtained with theory level HF/6-31G** and the atom names follows PDB conventions.

**Fig. 3:**
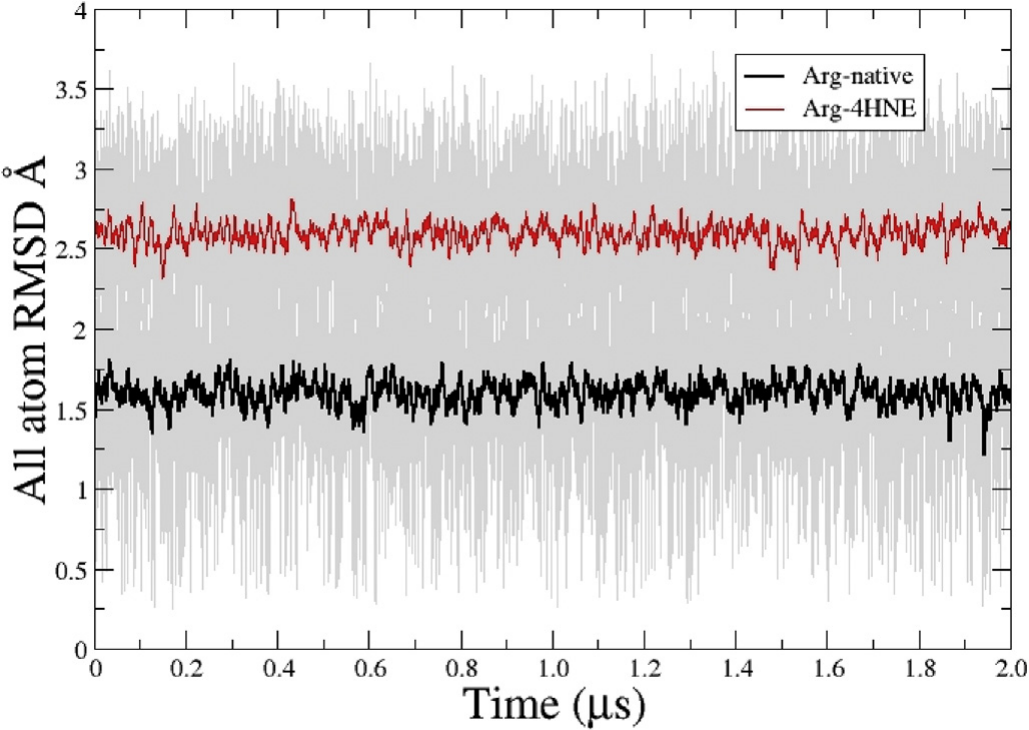
**Running average (using 40 frames) of all atom RMSD of unmodified and modified arginine with 4-HNE (non-standard) vs. time.** Raw data shown in the background. Unmodified Arg vs. Arg -4HNE. Black line corresponds to unmodified amino acid and the red line is to nonstandard amino acid. RMSD was calculated using an average structure of native amino acids as a reference.

The dataset included in this article consists of 3 Tables and 3 figures. In the Table 1 the dataset of partial charges assigned to Arg-HNE is shown. Table 2 contains the dataset with the information of new obtained parameters listed as coordinates file for 4HNE-arginine pyrrole adduct; while in Table 3 is summarized the comparative data of selected bond distances and angles used in the validation step. In Fig. 1, the workflow for preparing parameter files for 2-pentylpyrrole adduct is described; while, the optimized structure for the new non-standard residue obtained with theory level HF/6-31G** is presented in Fig. 2. The running average of all atoms RMSD for non-modified and 4-HNE-modified arginine is showed in Fig. 3. Finally, supplemental Prep and Frcmod files along to their instructions to perform molecular dynamics simulations with Amber package software of carbonylated proteins with 4-HNE -arginine 2-pentylpyrrole adduct are available in the Amber parameter database of Bryce Group: Computational Biophysics and Drug Design (http://research.bmh.manchester.ac.uk/bryce/amber/).

**Table 1:** Partial charges assigned to ARG-HNE.

Atom Name	Atom TypE	Partyal Charge	Atom Name	Atom Type	Partyal Charge
N1	n2	−1.318.600	H13	h4	0.171000
H2	hn	0.128500	C8	c2	−0.002400
C1	c3	1.179.600	C9	c2	−0.292500
H3	h1	−0.410500	H14	ha	0.172900
C2	c3	0.067400	C10	c2	−0.247500
H4	hc	−0.094100	H15	ha	0.137100
H5	hc	−0.094100	C11	c3	0.038800
C3	c1	−0.750100	H16	hc	0.015900
O1	o	−0.486700	H17	hc	0.015900
C4	c3	0.010700	C12	c3	0.027000
H7	hc	0.047500	H18	hc	−0.012800
H8	hc	0.047500	H19	hc	−0.012800
C5	c3	−0.431500	C13	c3	0.015700
H9	h1	0.137400	H20	hc	0.003900
H10	h1	0.137400	H21	hc	0.003900
N2	n3	−0.318000	C14	c3	0.037200
H11	hn	0.239300	H22	hc	−0.011200
C6	c2	0.631500	H23	hc	−0.011200
N3	n2	−0.904800	C	c3	−0.070900
H12	hn	0.374300	H24	hc	0.006300
N	na	−0.014200	H25	hc	0.006300
C7	c2	−0.175400	H	hc	0.006300

**Table 2:** New parameters assigned to ARG-4HNE.

A. BOND			
Atom Types	Kr	req	note
c3-ns	328.70	1.462	
ns-hn	403.20	1.013	
ns-c	427.60	1.379	
c3-nu	326.60	1.464	
nu-hn	404.60	1.012	
nu-c2	416.20	1.387	
C -ns	372.304	1.422	
c –N	282.464	1.512	

**B. ANGLE**			
**Atom Types**	**KΘ**	**Θeq**	**note**
h1-c3-ns	63.390	117.68	
c3-ns-hn	49.840	120.69	
ns-c -o	74.220	108.88	
c3-ns-c	45.800	123.05	
ns-c -c3	66.790	115.18	
hn-ns-c	48.330	117.55	
c -c3-ns	67.000	109.06	
ns-c3-c3	65.910	111.61	
c3-c3-nu	66.210	110.46	
c3-nu-hn	46.070	115.99	
c3-nu-c2	62.400	123.71	
h1-c3-nu	49.570	109.79	
nu-c2-n2	71.790	124.27	
nu-c2-na	72.891	111.07	
hn-nu-c2	48.590	115.09	
CX-C -ns	68.543	121.53	
o -c -N	81.645	120.93	
c -N -CT	62.307	137.45	
c -N -CX	68.788	119.90	
c3-c -N	68.543	121.53	
O -C -ns	81.645	120.93	
C -ns-c3	68.788	119.90	
C -ns-hn	54.751	114.31	
**C. DIHEDRAL**			

**Atom Types**	**Vn/2**	**γ**	**n**
c3-ns-c -o	1	180.000	2.000
c3-ns-c -c3	1	0.000	−2.000
c3-ns-c -c3	1	180.000	1.000
h1-c3-ns-hn	1	0.000	2.000
h1-c3-ns-c	1	0.000	2.000
hn-ns-c -o	1	180.000	−2.000
hn-ns-c -o	1	0.000	1.000
hn-ns-c -c3	1	180.000	2.000
c -c3-ns-hn	1	0.000	2.000
c -c3-ns-c	1	180.000	−2.000
c -c3-ns-c	1	0.000	1.000
hn-ns-c3-c3	1	0.000	2.000
c -ns-c3-c3	1	180.000	−4.000
c -ns-c3-c3	1	180.000	−3.000
c -ns-c3-c3	1	0.000	−2.000
c -ns-c3-c3	1	0.000	1.000
c3-c3-nu-hn	1	0.000	2.000
c3-c3-nu-c2	1	0.000	2.000
c3-nu-c2-n2	1	180.000	2.000
c3-nu-c2-na	1	180.000	2.000
h1-c3-nu-hn	1	0.000	2.000
h1-c3-nu-c2	1	0.000	2.000
hn-nu-c2-n2	1	180.000	2.000
**D. IMPROPER**			

**Atom Types**	**Vn/2**	**γ**	**n**
c -c3-ns-hn	1.1	180	2
c3-ns-c -o	10.5	180	2
n2-na-c2-nu	1.1	180	2
c2-cc-na-cc	1.1	180	2
cd-h4-cc-na	1.1	180	2
cc-cd-cd-ha	1.1	180	2
c3-cd-cc-na	1.1	180	2
C*-CN-CB-CA	1.1	180	2
NA-CA-CN-CB	1.1	180	2

**Table 3:**
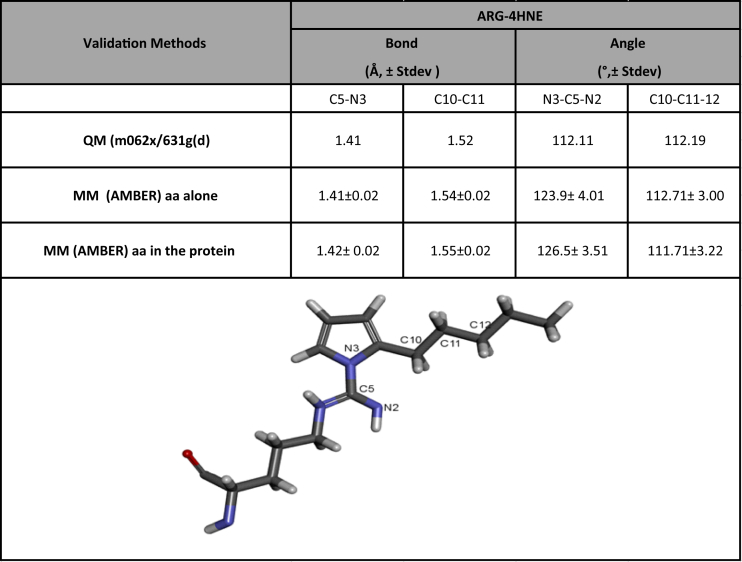
Comparison between selected bond distances and angles calculated from optimized nonstandard amino acids structures.

## 2 Experimental design, materials, and methods

### 2.1 Parameterization

Dataset of Gaff force field parameters were established for the non-standard amino acid Arg-HNE and its use for molecular dynamics simulations of proteins [1]. In the Fig. 1 is presented the framework for derivation of missing bond, angle and dihedral parameters. First, non-standard amino acid was constructed with GaussView 5, followed by full geometry optimization of the new structure using the Hartree-Fock level (HF/6 - 31G**) [2,3]. Next, assignment of charges, missing bonds, angles, and dihedral angles parameters were constructed with the antechamber and leap programs as included in AmberTools 16 [4]. Then, charges (Step 4) of the optimized structures were calculated using RESP method [5] and the partial charges assigned to individual atoms are listed in the Table 1. Missing bonds, angles, and dihedral parameters of 4-HNE modified arginine was established by homology, matching atom types automatically from the Gaff force field and using parmchk to generate the required force constants [4]. Dataset of new parameters assigned for the 2-pentylpyrrole adduct were consigned in frcmod files and they are summarized below in Table 2. Next, coordinate and topology files were created for each non-standard amino acid with the program leap.

This Arg-HNE was replaced on the proteins and the lacking parameters in frcmod files corresponding to peptide bonds, angle and torsions between the non-standard amino acids and the end nitro-terminus and the end carboxyl terminus of the nearby amino acids on proteins, were calculated using the program parmcal of Antechamber package. The improved frcmod file was loaded into tleap program from AmberTools16 to generate the libraries files (type lib files).

Finally, the optimized structure of 4HNE-arginine pyrrole adduct is showed in Fig. 2; whereas the new improved parameters were included into Table 2. There, bond parameters values are expressed as bond constants (**kr**) in *kcal·mol*^*−1*^*Å*^*−2*^; distance at equilibrium (**req**) in *Å*; angle constant (**kθ**) in *kcal·mol*^*−1*^*deg*^*−2*^*;* angle at equilibrium (***Θeq***) in *degrees*, dihedrals constants (***Vn/2***) in *kcal/mol* and dihedrals constants angles (**ψ**) in *degrees*.

From these datasets, the topology and coordinate of modified proteins were obtained. Hence, the applicability of the newly derived MM parameter, they were subsequently employed in 1 μs MD simulations of Arg-HNE as an amino acids treated following the methodology described by Refs. [1,6].

### 2.2 Validation

To test the generated structures from the modified arginine we performed MD simulations as described above using only the modified structure and compared selected bond distances and angles with structures obtained from DFT level of theory *m062x/631g* (d) (Table 3) [1]. Overall, good agreement between the data from high-level QM calculations and the generated AMBER structures were seen. Distance average error is in ∼0.02 Å whereas angle error is within ∼4 and 3 Å.

Data from the single modified amino acids were extracted from a 1 μs MD simulation using the same protocols describe before, comparisons were calculated using the DFT level of theory m062x and a basis set 6–31g.

### 2.3 Analysis of molecular dynamics trajectories of non-standard vs. standard amino acids

All atom root means square deviation analysis for unmodified and modified amino acids is presented in Fig. 3. Distance found in RMSD analysis for unmodified arginine was ∼1,5 Ålower than that found for ARG-HNE, which was ∼2.5 Å (Fig. 3). Differences observed fall into a range of 1 Å for RSMD comparisons among modified/unmodified arginine indicating that 4-HNE do not induce dramatically structural changes.
